# Artificial Human Milk

**Published:** 1883-04

**Authors:** William Henry Cumming

**Affiliations:** Atlanta, Ga.


ATLANTA

MEDICAL REGISTER.

VOL. II.] APRIL, 1883. [No. 7

### Original.

### ARTIFICIAL HUMAN MILK.

BY WM. HENRY CUMMING, M.D., ATLANTA, GA.

INTRODUCTION.

Of late years many farinaceous preparations have been recommended as good substitutes for the mother's milk. These we believe to be (so far as they are farinaceous) not only useless, but injurious; starch being utterly unfit for the stomachs of young infants. Food must be provided differing as little as possible from that which Divine Wisdom has prepared. We send forth this little work with the hope of extending the use of ARTIFICIAL HUMAN MILK, for this is the nature of the food here recommended, and the reason of its admirable success. In its composition it most closely resembles the natural secretion of the breasts in vigorous, healthy women; and it offers to the child all that he needs for growth, development, warmth and activity. A careful observation of its effects for thirty years has led to the conviction that it leaves nothing to be desired; and that, on this food, an infant may be reared with admirable results. And by this we mean that health, uninterrupted health, with vigor and energy of the bodily functions, may be regarded as the natural result of the use of this food. We mean, that disorders of the stomach and bowels do not follow its use, as they so often do that of mothers. We mean, that under its use, teething will be ordinarily a painless process; and that the teeth will be strong and durable. Believing that a large proportion of the sickness and death of infants is the result of insufficient and improper food, we feel sure that, by the use of this artificial human milk, the health and lives of tens of thousands might be annually preserved. We believe that, if generally used, the influence upon the next generation would be evident, in a visible increase of health and vigor.

Let it not be supposed for a moment, that we desire to release mothers from the plain duty of nursing their own children. Would that all mothers could furnish to their little ones this food so wonderfully adapted to their condition and wants. So far are we from wishing to see this duty more generally neglected, that we have given several pages of this work to show the great importance of maternal nourishment to the child, both before and after birth. We have taken the child under our protection from the moment of impregnation, and have endeavored to point out the existing evils and their remedies. We have shown his dependence on the mother's blood during his nine months' abode in the womb, and his almost equally complete dependence on her milk during one and a half or two years after birth.

But however painful the truth, it can not be denied that there exists a great and increasing degeneracy on the part of the women of this age and country. The number of those who are able to nourish their children fully, is exceedingly small; and we very much fear that in twenty years, it will be smaller still. It is time that this downward progress should be stayed, and that a movement in the opposite direction should begin. It is for this reason that we have devoted the first pages of this little work to a consideration of the growth of the child while in the womb, and to the evils arising from insufficient nourishment during that early and tender season of life. We have called attention to the evils of female education and training during the period of early womanhood, and sought to show their disastrous influence on infants, both before and after birth. This is a subject of great importance, not only to the physical, but to the moral welfare of women.

Must the wretched children be left to suffer and languish and die from insufficiency of natural food, when so good a substitute may be given? The answer to this question can not be for a moment doubtful. Every feeling of humanity urges us to save them, if we can, from their sad and painful end.

Nor let it be supposed that we intend to assert, that if children are well fed, they must of necessity be healthy and vigorous. Food is the first and highest want; if the child be not well fed, it can not prosper. But there are other conditions of health: warm clothing, pure air, sunshine, and exercise are of great importance. Our infants have many wants, and their successful nurture requires constant and enlightened care.

### THE BEGINNINGS OF LIFE.

Life begins in animals and plants in a point so small as to be visible only by the aid of the microscope. From this small beginning up to adult size, to the giant oak, to the mighty whale, all increase is gained from food. The germs of plants and animals, while yet invisible to the naked eye, are drawing from parts around them the materials needed for their growth. We find them increasing slowly and steadily, until their size is such that we may lay aside the instrument and observe their progress with our unaided vision. The circumstances amid which this growth takes place are exceedingly various, and the sources of nourishment differ in the various cases. But it is true of all, that materials similar to those existing in the future plant or animal, must be supplied and brought into contact with the germ; and that thus alone it can make progress and gain increase.

Concerning the growth of the human germ, we have no certain knowledge until conception has taken place. How great the action and influence of the male parent upon the future offspring, we do not know, but they cease from that moment. Thenceforth the germ continues for a time in complete dependence on the mother for food. It is to this period of human life that we shall confine our attention.

At the time of impregnation the germ is visible only by the aid of high magnifying power. It probably does not weigh the thousandth part of a grain. Let us trace its increase during the next nine months, while it derives its food solely from the mother's blood.

| | | | | | |
|---|---|---|---|---|---|
| At first its length is | | | 0 | and its weight | 0 |
| At the end of | 2 mos. | its length is | 1½ in. | " | 250 grs. |
| " | 4 | " " | 5 " | " | 1,200 " |
| " | 6 | " " | 9 " | " | 6,000 " |
| " | 8 | " " | 14 " | " | 30,000 " |
| " | 9 | " " | 19 " | " | 50 000 " |

Thus from a point invisible to the naked eye, the germ has grown to a length of 18 or 20 inches, and to a weight of 6, 7, 8, and even sometimes of 10 and 12 pounds.

How has this great increase been effected? By the use of suitable materials drawn from the mother's blood.

### THE VITAL FLUID.

The blood of our bodies contains the elements needed for their growth and activity. If the blood be properly constituted, it can supply all the demands of the system. But it often happens, either from insufficient food or from inability to digest it, or from unusual or excessive losses, or from some poisonous influence, that the blood loses this power of supplying the wants of the body. This change of the blood is soon shown by its effects. The strength declines, the organs fail to perform fully their appropriate duties, and sooner or later positive disease follows.

How important then to the unborn infant the good quality of his mother's blood. If he finds there all the materials necessary for his nourishment and growth, he will thrive and prosper during his life in the womb. But if these supplies are wanting, there is no other resource for him; he must languish and pine. In many cases this deficiency is so great that the child's life can not be maintained, and he dies in this first stage of his being. In less serious cases he continues to live, but it is not a life of vigorous growth and full development. The birth takes place, but there is not sufficient strength for the new circumstances of his condition, and after a few feeble breathings he ceases to live.

The differences among new-born infants in size and strength are very great. We find differences almost as great among the domestic animals at birth. Excessive labor on the part of the mother, or scanty food, will so dwarf and enfeeble the offspring that it either dies or is scarcely able to live. Starvation, toil, anxiety, and suffering among women often lead to death of the child before or just after birth.

Are we not right then in counseling mothers to nourish their children well *before* birth? A failure of food is probably more injurious to the child during this tender season than at any future time. And yet how few mothers care as they should for the helpless little ones thus committed to their charge. How many take no pains to promote their own health and vigor, by which alone they can promote the welfare of the child. How many are careless about food, exercise in the open air, sleep, and the other conditions of health. The inactivity so common among mothers at this period is most mischievous in its effects. While excessive exertion should be avoided, moderate exercise *in the open air* is of great importance to the well-being of mother and child. The sense of shame which keeps so many within doors during the last weeks and months of pregnancy, should be overcome by the higher feeling of maternal love, and the determination to promote in every way the true interests of the little ones so entirely dependent on their care.

### PHYSICAL TRAINING OF GIRLS.

These considerations should have an influence extending to all who have daughters yet in early youth. *Health and vigor are not the immediate results of earnest resolutions.* THEY COME FROM THE LONG CONTINUED SUCCESSFUL PERFORMANCE OF THE VARIOUS FUNCTIONS OF THE BODY. It is then too late to begin to attend to health when there is already another life depending on the mother. Years before this, a wise forethought should have seen this probable dependence, and made arrangements to meet it. A man having no visible means of supporting a family is deemed imprudent if he marry. A wise man will first provide the means of support, and then incur the obligation. And should not a woman exercise the same forecast, or rather, should not her parents do so? Of what avail is it to the unhappy unborn children that the father is rich in money, if the mother be poor in blood? Gold will not enrich the mother's blood, and they must suffer from starvation. Is it not plain, therefore, that every young woman should, if possible, possess health and vigor, that she may be able to nourish well the little ones that may soon be dependent on her? And how sad that so many trifle with health and strength, with the certainty of bringing sickness and suffering upon their future offspring.

And here we address ourselves especially to parents, for to them especially this duty belongs. If their young daughters are to become good mothers, their training must begin in early youth. How much care and money and time are expended on their mental culture, and in many cases with what result? The production of elegant, accomplished, feeble, sickly women, utterly unfitted for the very next stage of their existence. The majority of these young women are married within three years after quitting school; many of them before one year has passed away. Their very next duties in life are those of wives and mothers. How evident, then, that whatever else is neglected in the course of their education, the qualities needed in this next stage *should be secured.* And yet amid the many efforts made by parents and daughters during this season of study, how little care is given to the healthful and vigorous development of their bodily structure. Studies are carefully arranged and regulated, but what is done for health? Nay, more, is not the system usually pursued itself directly at variance with all the precepts of a wise hygiene?

### THE SEASON OF DEVELOPMENT.

The season of development for women is between thirteen and twenty years of age. During four or five of these years, the great changes needed for the transformation of a girl into a woman must take place, or they will not take place at all. IF THIS SEASON OF DEVELOPMENT BE LOST, THE EVIL CAN NEVER BE REPAIRED. THE DEVELOPMENT MUST TAKE PLACE THEN OR NEVER. How important then that girls should enter upon and pass through this season under most favorable physical conditions. Good food in abundance, a sound and vigorous digestion, sunshine, fresh air, bodily exercise, sleep; these are the conditions of health. Sedentary occupations, long continued or excessive mental exertion, late hours, insufficient sleep, are evidently most unfavorable to development. And yet, whether we look at the daughters of the rich or of the poor, does not this last sentence describe their state? The former, whether under the parental roof or in large educational institutions, give too much time to study, to reading, to music and the other fine arts, and are thus deprived of sunshine, fresh air, vigorous and invigorating exercise, and sufficient sleep. The appetite for simple, wholesome and nutritious food declines, the digestion is impaired, very little nourishment is obtained, and the great changes which properly belong to this season of life cannot fully take place. The framework of the trunk is not sufficiently enlarged, the chest remains too narrow, and the lower part of the body is not large enough to afford space for the organs of the abdomen. Nor does the frame alone suffer. The muscles become small and feeble by disuse, the blood loses its proper composition, the nervous system suffers, and languor and pain follow all unwonted exertion whether of mind or body.

In this general debility and disorder the generative apparatus has its full share. The growth and activity of these organs are hindered by the failure of the supply of necessary nourishment. The ovaries cannot properly mature the ova; and difficult, irregular and painful menstruation follows. The breasts, those fountains of life from which in after years starving nurslings will strive in vain to draw full supplies of their divinely appointed nourishment, now small and flaccid, bear witness to the general imperfection of the system.

It should never be forgotten that this whole apparatus, so important in the future career of a woman, can scarcely be said to exist before the age of puberty, so rudimentary is its condition. It is unfortunately true that in thousands and tens of thousands of cases this rudimentary condition is continued by reason of imperfect development, and disables multitudes of excellent women from fulfilling rightly their manifest destiny, the duties of their earthly mission. How often do these early derangements remain a source of suffering and disease through all the best years of life and even down to old age, if the victims do not sooner yield to their power. How many women remain barren through long years of married life, or bear feeble children, too feeble to be reared? What masses of suffering far above all computation arise from this source? How many households whose children are all in little graves? What tears and anxieties and anguish does each day witness: mothers bending in agony over their puny, feeble, diseased, dying but cherished children? Whence this amount of sorrow and mourning? We cannot account for all in this way, but we venture the assertion that most of it springs from the inability of mothers to perform their part in the work of reproduction.

The daughters of the poor suffer in the same way. Long confinement to sedentary in-door occupations, to needle-work, etc., deprive them of fresh air, exercise, and sunshine. The thousands who spend these years of youth in factories, breathing unwholesome air, and shut in from the invigorating influence of active and varied exercise, all fail to attain to that full and healthful development which this season of life should bring. The blood becomes impoverished. The pallor of the face, the languor of the movements indicate the low condition of this life-sustaining liquid. No sight is more painful to the physiologist than that of a long procession of these feeble, pallid, emaciated young women as they leave the factory for their noonday meal. And to such an one the question constantly presents itself, whether cheapness of cotton or woolen fabrics is not dearly purchased with the health and happiness of so many of the daughters of the land.

If these things be true, can we wonder that the function of generation is ill-performed? Is it strange that so many marriages are unfruitful, with this lack of development and general debility?

And when impregnation does occur, the season of pregnancy is most frequently a time of nausea and lassitude and suffering. How often do abortions (miscarriages) take place, impairing still further the general health! And in more fortunate cases, how often is the child at birth feeble and ill developed. The labors and sufferings and losses of child birth, seriously reduce the mother's strength. And if the child survives the perils of his entrance on this second stage in the great journey of life, how unlikely is it that his mother will be able to supply his new wants, demanding much more bodily energy than has been called for in the season previous to his birth.

### MILK FOR BABES.

The second stage of the child's life is at least twice as long as the first, lasting one and a half or two years, extending from birth until the first set of teeth are fully formed. This is a natural division of human life, and it is marked by the use of a peculiar nourishment. Having left the body of the mother, the child no longer draws his supplies directly from her blood, and yet his dependence upon her is scarcely less complete than in the previous condition. The milk is derived from the blood, and resembles it in many important respects. It contains all the materials entering into the structure of the child, and is thus well fitted to promote his rapid and healthful growth. It supplies him also with the fuel needed for the production of vital heat, and thus enables him to maintain a steady temperature unaffected by the changes in the atmosphere around him.

These are the general facts concerning milk; but for greater precision, let us consider the case of a vigorous, healthy, new born child, that we may understand his wants and learn the adaptation of milk to his peculiar condition. In order to set forth more distinctly the first want of a child, let us suppose him born in the midst of a rigorous winter.

WARMTH.

The child must be kept warm, or he will suffer and languish and die. How shall this be done? "By wrapping him up in flannel," some would say. But will this do? Take a bottle of boiling water and wrap it in as many folds of flannel as you will, and put it in a warm bed, and how long will it retain its heat? In twelve hours it will only be tepid, and in twenty-four hours it will have become cool. The reason of this is, that there is no internal supply of heat, and so the temperature steadily though slowly declines, until it reaches that of the surrounding air. Treat a child in the same way, and you would have the same result, if he did not breathe. Does not everybody know that a dead body soon becomes cold? But if the child breathes, he will not so rapidly lose his temperature; if he breathes fully and well, his temperature will be maintained without loss. And how does breathing enable him to keep up his heat? It is certainly not the entrance of cold air into his body and the issue of warm air that will keep him warm. Thus considered, breathing is a chilling instead of a warming process. *Breathing is an internal combustion.* It is the burning of oil and other substances contained in the blood. This combustion takes place in all parts of the body, and thus the heat is uniformly distributed. The object of breathing is, first, to introduce air to support this combustion, and second, to carry off the produts of the combustion itself. Now, this combustion implies a consumption of fuel. This fuel is oil. To maintain the vital heat, there must be a constant supply.

BUTTER—THE INFANT'S FUEL.

How then is the child to be kept warm? By supplying him freely with oil. And how is this done? By the butter of the milk which he takes.

How much butter does he need?

In order to answer this question, we must ascertain how much is furnished to, and taken by a well fed and healthy child. Human milk has the following composition:

| | | | | |
|---|---|---|---|---|
| Butter, | 20. 76, | or | $20\frac{3}{4}$ | thousanths. |
| Casein | 14. 31, | or | $14\frac{1}{3}$ | " |
| Sugar | 75 02, | or | 75. | " |
| Water, | 889.88, | or | 890 | " |

A vigorous child, three months old, takes about $3\frac{1}{2}$ lbs. daily. This will give for the last nine months of the first year 955 lbs. To this add 250 lbs., as the quantity consumed in the first three months, and we have 1205 lbs. as the quantity of milk taken during the first year. But we have seen that this milk contains $20\frac{3}{4}$ thousanths of butter; 1205 lbs. contain, therefore, 25 lbs. This amount of butter is needed by the child during the first year.

Some will think this quantity enormous, and will say: "Why, the child does not weigh more than 25 lbs. at the end of the year." That is true, and it serves to show plainly that the butter has been *expended* by the child. It is his fuel, and a child needs about 25 lbs. to keep him warm during the first year. And with this consumption of butter, the child *is kept warm.* Compare the warmth of a well-fed, healthy child with that of an ill-fed and feeble one. The hands and feet and legs and arms of a vigorous child seem about as warm as the back and breast; while in a feeble child the extremities are usually cool.

BUTTER—THE FOOD OF THE NERVOUS SYSTEM.

But the butter has another use, which must now be noticed. Active exertion is necessary for the growth of the muscles of the body. We see this among adults. Look at a blacksmith's right arm; what has made it so muscular and strong? Exercise. How do you know this? By comparing the right arm with the left. The latter may be muscular too, but it is far smaller and weaker than the right. Now, young infants have very little muscular power. They acquire a great deal during the first year, and they acquire it by constant exercise. Watch a healthy infant after the age of three or four months. He is never still when awake; he is always doing something Now, this constant activity of mind and body demands great nervous energy, and this requires that the nervous system (brain and nerves) should be well fed. The brain and nerves are of peculiar structure, containing a peculiar material, and this material must be constantly supplied in the food, or the nervous power will decline. This peculiar material is a phosphuretted oil named Lecithin. It exists in great quantity in the yolk of the egg, and hence (from the Greek word Lekithos) its name. The butter contains about one twelfth of its weight of this oil, and thus an infant, during the first year, receives (in 25 lbs. of butter) about two pounds.

We thus see that the butter furnishes fuel for the production of vital heat, and material for the support of nervous energy. And it is worthy of remark here that the fuel without the nervous energy would not keep the child warm. Every one sees that it is not *the fuel itself* but *its combustion* that warms us. A man might freeze to death with his cellar full of coal if he did not burn it. So if you should furnish to a child oil without Lecithin, he could not keep warm. He must not only have oil enough in his blood, *but he must burn it.* And how is this combustion effected? By breathing freely, so as to introduce an abundance of air into his lungs, and by a lively circulation of the blood through the lungs, so that the oxygen of the air may be freely taken up and carried into every part of the body, there to unite with the oil and give forth the required heat. Now, this full play of the lungs and heart requires nervous energy, and this nervous energy requires for its support a supply of Lecithin. The Lecithin blows the fire; if the blast ceases, the fire will decline and go out. And it is a remarkable fact in the case of starving animals, that as soon as the nervous system loses a very small proportion of its weight, the animal becomes suddenly cold and dies.*

CASEIN—THE FOOD OF THE TISSUES.

We have seen that the child receives in 1205 lbs. of milk about 25 lbs. of butter; this keeps him warm and supplies nervous energy. This is not all that he needs, however. He must grow. And this brings us to the second great ingredient in milk; Casein or Cheese. Four-

*Appendix—Lecithin.

teen and one-third thousandths are found in human milk. In the first year, therefore, the child will receive (in 1205 lbs. of milk) 17¼ lbs. of casein, and it must be remembered that this is *dry* casein. Now, a child during the first year gains only 15 or 18 lbs. in weight and these 15 or 18 lbs. contain not more than 2 lbs. of dry solids, the rest consisting of water. So that of the 17 lbs. of dry casein 15 lbs. are not employed in the growth of the body; by this we mean that only two pounds of the 17 are retained within the body and increase its weight; the other fifteen have been swallowed, digested, used, and carried away. What is the use of this waste of food? There is no waste: it is all well employed. Does a laboring man waste several pounds of food every day because he gains no weight? The food does not make him grow, *but it enables him to work.* No exertion can be made without the loss of some portion of the organ making the exertion. If a man works hard, and gets insufficient food his muscles decrease in size and strength. So a child, ever in motion when awake, requires a great deal of solid food, and this he gets from the casein or cheese of the milk. He uses 15 lbs. in muscular exertion, and only 2 lbs. in actual growth.

### BONE-EARTH IN CASEIN.

One of the most important articles of food, during the infancy of a child, is phosphate of lime or bone-earth. The yielding bones of a new-born infant, must become much firmer and stiffer before they can bear the weight of the body. The distortion of the bones in rickety children arises from a deficiency of bone earth in their bones. The teeth, too, need this same material for their growth. With a full supply of good food, the growth of the teeth is an easy and painless process. But how many children suffer from teething. How few there are who go through this process without pain. And in how many cases do the teeth decay before the child is five years old. The cause of all this suffering is the deficiency of bone-earth wherewith to make the teeth. If this material be fully supplied, (as it is in well-fed children), the teeth give rise to little or no inconvenience. Now, one of the most interesting peculiarities of casein is that it contains a very large proportion of bone earth, so that a child abundantly supplied with casein will obtain from it enough bone-earth for all his wants, and may make teeth and bone without difficulty. And it is thus explained to us why starch-fed children suffer so much while "teething." The starch fails to furnish the bone earth, and without this *the teeth can not be made.*

SUGAR.

The child obtains in the first year's milk about 90 lbs. of sugar. On account of its chemical composition it is generally believed that it cannot directly nourish the tissues of the body. It is known that it acts as fuel, uniting with the oxygen of the air and giving forth heat as the result of this union. By many this is supposed to be its only use. It is perhaps best to say that it is not yet certainly known to have any other use.

FUEL-FOOD.

It thus appears that 25 lbs. of butter and 90 lbs. of sugar are required as fuel during the first year of a child's life. On account of the superior heating power of oils, the 90 lbs. of sugar are equivalent as fuel to 36 lbs. of butter. The child then uses the equivalent of 61 lbs. of butter for heating purposes in one year. This is as though a grown man weighing 150 lbs should use 500 lbs. of butter annually, or 1½ lbs. every day.

How shall we account for this seemingly disproportionate demand for fuel in a child? How is it that a child needs twice as much fuel-food as a man, their respective weights being considered? The explanation is very simple. The infant's external surface is twice as large for his weight as that of a man. A man's surface is equal to 2500 square inches. A new-born infant's weight is about one twentieth of a man's. If his surface were in the same proportion, it would be 125 square inches. As his length is about 20 inches, this would give him less than seven inches of girth. The fact is, that a child weighing 7 lbs. has 250 square inches of surface, and, therefore, twice as much for his weight as an adult. The latter has 18 inches of surface to every pound of weight; the child has 36 inches for the same weight. As the loss of heat is proportioned to the extent of surface, the child ought to have a double supply to compensate for his double surface. And this double supply is furnished by the milk.

WATER.

The water in the milk is in very large quantity; almost nine-tenths of the whole. A child drinks about 1072 lbs. a year, or on an average almost three pints daily. With this quantity, equivalent to three gallons daily for a full-grown man, the healthy infant has enough to keep him from suffering from thirst. As the child's external surface is relatively twice as great as that of an adult, the cutaneous perspiration consumes a much larger quantity of liquid.

ANNUAL CONSUMPTION OF MILK.

The quantity of milk required by the child varies greatly at different periods. During the first ten days, the child does not take more than from 1 to 1½ pints a day. Before the end of the first month, he requires more than a quart daily. This demand increases until at the age of three months 1½ or 2 quarts are taken. From this time on there is no marked increase in the quantity taken. The quantity remains the same, while the quality changes.

The milk furnished to the new born infant is so peculiar in its character as to have received a special name: it is called Colostrum. As time advances, it gradually loses these peculiarities and becomes the ordinary milk.

DRY SOLIDS.*

We have seen that the child consumes in the first year 1200 lbs. of milk. This milk contains 132 lbs. of *dry solids* namely, butter, 25 lbs.; casein, 17 lbs; and sugar, 90 lbs. Now, a woman of feeble digestion can not furnish this great quantity of dry solids. She may perhaps furnish 1200 lbs. of a liquid called milk, but the solids will be fearfully deficient. Now, these solids are needed by

***Appendix—Dry solids.**

the child; the water will neither warm nor feed him. If the solids be deficient, he can not grow and thrive And yet many (may we not say most?) mothers are furnishing milk very deficient in these important materials. And, that this is true, will be made very apparent from the following statement: A strong, healthy, fat woman almost always loses weight while nursing a child, although supplied with as much wholesome food as she can eat. Even while eating thus largely, and digesting well her food, she can not do more than supply her child. What then are we to expect from a feeble woman, whose digestion scarcely suffices for the support of her own body? If she could supply this large amount of food to a nursling, could she not have taken better care of herself? A woman who can not digest large quantities of food can not be an efficient and successful nurse. A laboring man does not consume more food than a woman who is fully nourishing a child.

A comparison of the quantity of milk furnished by a cow with that required by a vigorous child, may be here profitably introduced. We have seen that a child needs 1,200 pounds in the first year. We will suppose the mother to weigh 132 pounds. She has then to give nine times her weight in milk in one year, and this milk must contain her weight (132 pounds) of dry solids. Let us take a cow weighing six times as much, or 800 pounds; she must give 800 pounds of dry solids in a year. This will require 6,154 pounds of milk in a year, or 7 quarts daily. This is a very large quantity for a cow to give as a daily average for a whole year.

It follows from this, that in fully nourishing a child, a woman must give as much milk in proportion to her weight as a good cow. And yet how few mothers have powers of digestion at all adequate to such a result. A large number of mothers give less than one-half of this amount; their children must therefore suffer unless a good substitute can be procured.

With this large supply of butter, the child is not excessively fat; this shows that there is no excess in the supply of oil. Let us then consider it as a settled truth that the child *needs* 25 lbs. of butter during the first year. And let us not be surprised if, with a smaller allowance, he becomes pale and languid and cold. The importance of a high temperature to bodily vigor must not be forgotten. Without an abundance of vital heat, there can be little vital energy.

We are probably within limits when we say that most of the mothers of this country fail to supply fully their infants with milk. Some fail almost from the very beginning; some do well for two, three, or four months; a very few continue to the end of the first year, and not one in fifty keeps up a good supply for fifteen months. And this leads us at once to consider the length of time during which a woman should furnish milk in large quantity to her child.

HOW LONG SHOULD MILK BE GIVEN?

This is a most important question. How long does a child *need* milk as his sole or *principal* article of food? How old must he be before being weaned? There are three different sources of information on this subject.

And first, let us look at a vigorous woman, one who evidently performs this duty well. We shall find that such a woman gives milk for fifteen or eighteen months; some of them go beyond this period.

Secondly, let us look at other animals, and observe at what stage of development they cease to depend on their mothers for food. To those who are acquainted with the condition of young animals of different kinds at birth, it is scarcely necessary to remark that they differ greatly in this respect. The calf, the kid, the lamb, are well-known instances of the highest development. Next comes the colt; after him the pig, and lower down, the puppy, the kitten, the rat come into the world in a much less advanced state. The human infant is at birth far inferior in development to the first named animals, and somewhat superior to those last mentioned. Let any one familiar with the condition of new born calves say at what age the human infant attains an equal development. He will probably fix upon the age of nine or ten months in the case of healthy, vigorous infants. Feeble children frequently fail to reach this advanced state in 12, 15, 18, or 24 months. Those who rear cattle know that the calf receives milk for five or six months, and they know too, that a calf thus fed is a far finer animal than one deprived of this natural food. Now, let us suppose a human infant nine months old equally advanced with a new-born calf, and we shall have at the least six months longer to supply him with milk. But we must remember that the calf belongs to a comparatively short-lived race, becoming an adult at the age of 4 or 5 years, and dying of old age at 25 or 30; whereas the human infant belongs to a long-lived race, not becoming an adult before the age of 20, and living 70, 80, 90, or 100 years. The stages of a calf's life are, therefore, only one-fourth as long as those of a child, and hence five months of lactation for a calf would represent at least 20 months for a child. Add, then, 20 months to 9, and we have 29 months, or nearly $2\frac{1}{2}$ years as the age at which a child should be weaned.

But, on the other hand, it must be remembered that the usual food of the calf is more difficult of digestion than that given to the human infant, and, therefore, it is not necessary that the child should be so well developed as the calf at the time of weaning.

Let us, in the third place, consider the apparatus for mastication in the child, that we may learn at what age he is prepared to have solid food. The first set of teeth (milk teeth they are called) are in vigorous infants usually complete at the age of $1\frac{1}{2}$ or 2 years. Twenty-one months would probably be a safe age to fix upon for vigorous, well-developed children. At that age they may chew with success, and, thenceforth, may be considered able to dispense with milk altogether.

It would probably be a fair inference from these considerations, that children may be wholly weaned when they have completed their first dentition; that to vigorous children 18 months old, or having 16 teeth, good food (other than milk) may be given in gradually increasing quantity; and that to slowly-developed children (no matter what their age) no other food than milk should be given until 16 teeth are out.

HOW TO GET SO MUCH MILK?

But here the question will be asked: "Whence is the milk to be obtained?" And this brings us to the second part of our subject--the consideration of the effects of insufficient food upon infants, and the remedy for this deficiency.

We have spoken of the amount of food needed by an infant. We have said that he needs 3½ lbs. daily after the age of three months Before that age he needs from 2 to 3½ lbs. This statement may be readily verified by weighing a child before and after feeding. If three hours have elapsed since the last feeding, it will be found that he weighs half a pound more after taking as much milk as he wants. In this experiment, both mother and child must be vigorous and healthy A child requires six or seven such doses daily, or from 3 to 3½ lbs. Many of the mothers who read this, know that they have never supplied this quantity, and have never fully satisfied the wants of their infants. They have attempted to remedy this deficiency by giving to their little ones bread, panada, corn starch, barley, arrow-root, or some other farinaceous food, mingled, perhaps, with a certain quantity of milk. But they have not thus succeeded in rearing healthy, vigorous infants. The children have suffered from colic, constipation, diarrhœa, or some other disorder of the bowels; the teeth have given rise to pain and fever, and the fears of the parents have been often excited by alarming symptoms. It is true that children thus fed do not all die; but many do, and this not from any distinct, clearly-marked disease, but from debility and starvation. The fatal disease called cholera infantum, which sweeps off so many tens of thousands during the three hot months, is not common among well-fed and thriving infants.

We propose to parents, in this work, the use of food nearly if not quite equal to the best quality of human milk, and far superior to that furnished by most mothers. We believe it able, not merely to sustain life, but to promote a vigorous, steady, healthful growth. We believe, that if generally used, it would greatly diminish the annual mortality among infants, and would exert a marked influence on the health of after years. It furnishes, when properly administered, all that an infant needs for growth, activity and health.

By reference to what has been said concerning milk, it will be seen that a substitute for the natural food of the infant must have very peculiar properties. No article in the vegetable kingdom with which we are acquainted bears any resemblance to milk in its internal composition. We cannot imitate human milk by means of flour, starch, or any other substance of the kind. Nor can we do it with any substance but one that can be found in the animal kingdom, and that one is *Milk*. The milk of animals bears a greater or less resemblance to human milk, and it is among them that we must find our substitute for it.—The animal milk most readily obtained is that of the cow, and practically we can get no other in most cases. The milk of the goat, which is sometimes used for this purpose, is less suited to meet our wants, and, therefore, to the milk of the cow we will exclusively direct our attention.

### COW'S MILK.

But the objection will at once be made by many readers, that cow's milk has been tried by thousands without success, and this when the milk has been pure and fresh. This is freely admitted, for in the country parents often fail when they attempt to rear infants on cow's milk. Many mothers in the country, where cow's milk is abundant and cheap and good, use starch or arrow-root or barley rather than milk, because the milk disorders the infant's stomach. Let us explain this failure. The various animals furnish milk of various kinds to suit the various degrees of development of their young at birth. The milk that is adapted to a new-born calf is not suitable for an infant. It must be modified, or else it will do harm rather than good. Every one knows that an infant can not bear pure cow's milk, and every one adds more or less water to weaken it.* Yes, there is too much cheese in cow's milk, and the child cannot digest it. There is

* Appendix.—Intestinal Irritability.

almost three times as much as in human milk, as will be seen by looking at this table:

| | | | |
|---|---|---|---|
| In a thousand parts of cow's milk there are of | Butter, | 38.59 | parts. |
| | Casein, | 40.75 | " |
| | Sugar, | 53.97 | " |
| | Water, | 866.69 | " |
| | | 1000.00 | |
| While in a thousand parts of human milk there are of | Butter, | 20.76 | parts. |
| | Casein, | 14.34 | " |
| | Sugar, | 75.02 | " |
| | Water, | 889.88 | " |
| | | 1000.00 | |

Therefore, to reduce the casein of cow's milk (40.75) to the proportion found in human milk (14.34), we must add 1 4-5 times, or nearly twice as much, water, that is, to ten (10) parts of milk we must add eighteen (18) parts of water. Now, milk thus diluted would not be likely to disagree with a child, that is, it would not produce derangement of the stomach and bowels; but after a while we should find that the child was not thriving on this diet; and by examining the table we shall see the reason. By watering the milk so largely, we have diminished the proportion of butter as well as of casein. Now, by turning back to the paragraphs on the uses of butter, we shall see that the child cannot dispense with the proportion of this oil. By diluting the milk with 1 4-5 parts of water, we have reduced the butter from 38.59 to 13.58 thousandths. This is just about two-thirds of the proportion of butter existing in human milk, which is 20.76 thousandths. Suppose that a child were to take this milk for a year, instead of 25 pounds of butter, he would get only 16½ pounds. But we have seen that he *needs* 25 pounds in order to thrive and prosper. He must, therefore, languish on this short allowance.

ARTIFICIAL HUMAN MILK.

To those at all familiar with the elements of arithmetic, it will be evident from the foregoing table that dilution of ordinary cow's milk will never enable us to imitate the composition of human milk. In human milk the butter is to the casein as 20.76 to 14.34, or as 100 to 70. In cow's milk the butter is to the casein as 38.59 to 40.75, or as 100

to 105. Dilution will never change this relation of the two substances to each other; therefore, dilution will be vain. But if we could get cow's milk of the following composition: Butter, 54; casein, 38; sugar, 52; and water, 856; and should add to it sugar, 142; and water, 1458, we should have a milk of the following composition: Butter, 54; casein, 38; sugar, 194; and water, 2314

This would give, when divided by 2.6, (to reduce it to thousandths), butter, 20.77; casein, 14.61; sugar, 75; and water, 889.62.

| | | | | |
|---|---|---|---|---|
| Butter 54 | = | Butter 54 | ÷2.6= | Butter 20.77 |
| Casein 38 | | Casein 38 | | Casein 14.61 |
| Sugar 52+ 142 | | Sugar 194 | | Sugar 74.62 |
| Water856+1458 | | Water2314 | | Water890.00 |

Compare this result with the composition of human milk previously given, and you will find the difference unworthy of notice.

But can such milk be obtained? Yes; in two ways

The first way is by taking *the upper third* of cow's milk that has *stood* for four or five hours. This upper portion contains about fifty per cent. more butter than the ordinary milk of the cow. To obtain one quart of this milk, set *three* quarts of milk aside, and at the end of four or five hours, remove the upper quart.

Another, and in warm weather a better way, is by taking milk from the latter half of that given by the cow. The first part of the milk taken from the cow contains very little butter; the last part (called in the country "strippings") is very rich. The former half of the cow's milk contains about 21 thousandths of butter; the latter half contains 56. If the cow gives eight quarts at a milking, use milk taken from the last four quarts. Milk the first half into one pail, and set it aside, and then taking another pail, milk the other half into it, taking care that the cow be milked dry, for the last portions of the milk are by far the richest.

This milk, when diluted with 1½ parts of water, and properly sweetened, resembles ordinary human milk.

### CHOICE OF A COW FOR THIS PURPOSE.

Health and vigor are the most important qualities in a nursing cow. She should be in the prime of life—from four to ten years old—free from disease, and evidently in fine health. It is not important that she should give a great quantity of milk. Her calf should not be less than two weeks old; and when the calf is four or five months old, she must be given up, and another cow with a young calf obtained, if the best results are desired. She should have good pasture in summer, and in winter an abundance of good hay, and of water. No slops of any kind should be given to her; no pot-liquor, no swill, no brewer's grains, no refuse vegetables, nor turnips, nor carrots, nor potatoes, nor indeed anything but hay, and salt and water. With this mode of feeding, the milk will be of excellent quality, though the quantity will be far less than if the other articles of food were used. Grass is the cow's appropriate food, and her milk may be greatly changed by the use of these various articles of diet.

The cow should be well housed in cold weather, in a clean, large, and well ventilated stable. She should be allowed to go out for exercise whenever the weather will permit, but she must not be exposed to cold rains. Indeed, her health should be cared for in every way. If a cow is too much exposed to the cold, there will be very little butter in her milk.

Our experience teaches us that, four or five months after calving, the cow should be given up, and milk obtained from a cow having a young calf. This change to younger milk we have always found advantageous to the child. It is perhaps connected with a falling off in the quality of the milk due to a new pregnancy of the cow, but of this we are not sure.

VARIOUS DILUTIONS FOR VARIOUS AGES.

We have said that the mother's milk has, for the first two weeks after the birth of the child, a peculiar composition and a special name. It is called "Colostrum." From its first appearance, the milk gradually changes until it becomes ordinary milk. Our mode of feeding involves the imitation of these several qualities of milk. We call our substitute *artificial human milk*; let us begin by preparing artificial colostrum. To do this, we must use milk containing much more butter than that already described.— We must take the upper *eighth* instead of the upper *third* of milk that has stood for four or five hours. Thus, in order to get half a pint of this very oily milk, we must use two quarts of milk, and skim off carefully half a pint. Or we may obtain the same result by using the last tenth of the milk furnished by the cow. Thus, if a cow gives five quarts at a milking, the last pint will be sufficiently rich for this purpose. This milk must then be diluted with water, as will be seen in the following schedule.

It will be seen from this schedule that the milk is made more nutritious as the child advances in age. This schedule is arranged to suit the wants of vigorous children.—But in using it, great care and skill are needed. Regard must be had not to the mere *age*, but to the *condition* of the child One child goes on prosperously to the age of six months; he is in every sense six months old. The dilution directed for that age will suit him exactly.

Another, during his third fourth and fifth months, is sick and feeble. On learning these facts and observing his condition, we should say: "This child was born, it is true, six months ago, but his time has been ill-spent, he has not made the usual progress; we must reckon him as only four or five months old." Here the counsel of a physician is needed. If to a feeble, ill-developed child we give the milk suited to his *age*, we commit a great error, and shall fail in our experiment. It is indeed always safer to begin with milk somewhat more diluted than the child's age indicates, and to increase its strength, as the case will permit. It is less hurtful that the food should be insufficient than that it should be indigestible.

As this is a point of great practical importance, we will dwell upon it a little longer. Mothers usually consider these dilutions excessive, and are very impatient to put their children on stronger food. We repeat then that this schedule is arranged to suit the wants of *vigorous, healthy* children; *it will suit no others.* If the child has progressed steadily and rapidly, without intermission from sickness or injury of any kind, the dilution here directed will meet his case. But if from improper or insufficient food or from sickness, or from any other cause, he has fallen behindhand, a dilution suited to a younger child would be better for him. If the milk be too strong for him, indigestion will follow, and the child will lose instead of gaining strength. Particles of casein will pass through his bowels unaltered, irritating as they go. In such cases a younger quality should at once be substituted. A feeble child of nine months of age will probably require the food suited to a vigorous child of six months.

SIGNS OF DEVELOPMENT.

One of the best guides in ordinary cases is the development of the teeth. We here subjoin a schedule showing the age at which, among vigorous children, the teeth may be fairly expected. It is not, however, pretended that this order of development is, among vigorous children, invariable.

| | | | | | | |
|---|---|---|---|---|---|---|
| A vigorous child at | 7 or 8 | months | of age | will have | 2 | teeth. |
| | 9 or 10 | " | " | " | 4 | " |
| | 11 | " | " | " | 6 | " |
| | 12 | " | " | " | 8 | " |
| | 15 | " | " | " | 12 | " |
| | 18 | " | " | " | 16 | " |
| | 21 or 24 | " | " | " | 20 | " |

Notable exceptions to this rule will sometimes be observed. Children apparently healthy and strong are sometimes very slow in teething, but in most cases the teeth indicate the condition of the child.

The anterior fontanelle (V. Note B) is often closed at twelve months of age. In almost all well-developed children it is closed at or before the sixteenth month.

By making these observations concerning the growth of the teeth and the size of the fontanelle, we may usually determine what dilution will suit the child. A simple rule, which may be used by any careful mother, is to use weaker milk, if any curd is observed to pass through the bowels undigested. If the milk is too strong for the child, this result will soon be apparent.

If a child using this food become sick, it is well to diminish at once the strength of his food to that of a child three or four, or even sometimes (in severe cases) six months younger. In such cases the physician alone can decide wisely. It is a marked advantage of this artificial lactation, that in sickness no new *kind* of food is required, but the *same kind*, of *different strength.*

The quantity of butter may sometimes be advantageously increased. It will sometimes happen that while using milk prepared according to the foregoing directions, constipation will occur. An addition of butter to the milk will here be useful. There are two or three ways of doing this. In cold weather (or in hot weather, if the milk be kept in ice), the milk may be allowed to stand one or two hours longer before the upper third is removed. The upper third will, in this case, of course contain a larger proportion of butter. Or the upper *fourth* may be taken instead of the upper *third*. Thus, in order to get a quart of very rich milk, use four or five instead of three quarts, and remove the upper quart. The same result may be obtained by taking milk from the last *third* of that given by the cow. This is about 25 per cent. richer than the latter *half;* containing about 65 thousandths of butter. Thus, if a cow gives six quarts at a milking, take milk from the last *two* quarts instead of the last *three.*

DILUTION AND SWEETENING.

The water used in diluting milk should be as pure as possible; hard water must be unwholesome for infants. It should not be heated. The sugar should be good loaf sugar; brown sugar should not be used. The sugar may be added by taste. Care must be taken that the milk be made only a little sweeter than that of the cow. If too much sugar be used, the child will take less milk, being apparently cloyed.

The milk should be prepared twice a day in warm weather, unless it be kept in ice. In cold weather it may be prepared once a day.

If there be any doubt about the cow's being fully supplied with salt, it would be well to add a little to the infant's food. It would be much better, however, to supply the cow herself. With a deficiency of salt we must look for an impaired digestion.

MODE OF ADMINISTRATION.

The milk should be given by means of a bottle. Suction is the natural mode, and has several advantages. By this mode we can give it at a more uniform temperature. We can also thus (by the efforts and movements of suction) secure a full flow of saliva, which is needed for the digestion of the food. The child can lie down while feeding, either on his bed or in his mother's arms. There is less danger of his throwing up any part of the milk; his position need not be changed, and he will sink to sleep quietly, if his time for sleeping has come.

We have said that a child after three months of age takes half a pint or eight fluid ounces at a time. A bottle of this size will be required. The kind we prefer is one of elliptical form. Bottles having letters blown in the glass are cleaned with great difficulty, and should not be used. It is well to anneal the bottle by putting it in cold water, heating the water gradually until it boils, letting it boil for two or three hours, and then leaving it to cool very slowly. A bottle thus prepared is not likely to crack when placed suddenly in hot water when cold, or in cold water when hot.

A goose quill of moderate size, and 1¾ inches long, rolled up in a strip of Swiss muslin, makes the best artificial nipple with which we are acquainted.

The muslin should be moderately fine. A piece fifteen inches long and six wide is required. It should be folded so as to be 15 inches long and 1½ wide. The quill should be rolled in it so as to form a tightly-fitting stopper for the bottle. One end of the quill should be near, (but not quite at) the outer edge of the muslin. The frayed edges of the torn muslin must be turned in so that the threads may not get into the child's mouth. The stopper thus prepared must be forced into the bottle tightly, leaving two-thirds or three-fourths of an inch projecting from the neck.

The bottle, quill, and muslin should be frequently washed with soap and water. Without cleanliness there can be no success. The milk when prepared must be kept in a cool place, and pails, pitchers, pans, and cups must be clean and sweet.

The milk should be given at regular intervals. Many nursing mothers are in the habit of putting an infant to the breast whenever it cries. Where the supply of milk is insufficient and the child consequently always hungry, this may be necessary, but in artificial feeding there is no excuse for such conduct. The child should have at each time as much as he wants. He will then need no more for 3 or 3½ hours. The stomach will thus be left at leisure to accomplish the work of digestion. There is no rule more important than this in the artificial rearing of children. Success is impossible on any other plan. The child should be early trained to pass six or eight hours at night without being fed. This habit may be formed frequently before the age of two months.

Care should be taken that the child should not be bathed soon after being fed. The better plan is to bathe him 2½ or 3 hours after the last meal.

The temperature of the milk when given to the child should be about 100°, or about the temperature of the human body. The mother can soon train herself to recognize this temperature by feeling. By applying the bottle to the cheek, the heat can be most easily determined. The use of a thermometer may be at first advisable. The best mode of heating the milk is by putting it, when cold, into the bottle and placing the bottle in a bowl or cup of hot water. A little practice and care will soon enable the mother to manage this department very satisfactorily. It is important that the temperature of the milk be regulated with exactness. It will not do to depart much from the temperature prescribed.

The milk should be taken slowly; ten minutes should be given to each meal. By a proper arrangement of the quill and muslin, the flow of the milk may be controlled. The stomach will not then be too much distended, as the liquid part will be quickly absorbed.

### LENGTH OF ARTIFICIAL LACTATION.

One of the advantages of artificial lactation is that it may ordinarily be continued as long as necessary. Mothers are frequently compelled to wean their infants long before the infants should be weaned, and in such cases we advise them to adopt this food. They may thus promote the vigorous and healthful development of children, likely otherwise to suffer for want of proper nourishment. We have said that children should depend principally, if not solely, upon milk until the age of two years, or until they obtain their first set of teeth. Some feeble children will need milk up to the age of two and a half or three years.

Such children often suffer for want of suitable food. What, in all probability, can be better for them than this food of nature so wonderfully adapted to their wants?

We advise, then, that children should rely exclusively on milk until they have sixteen teeth fairly developed. From that time on, food of other kinds, eggs, bread, puddings, may be given in small quantities, and of the best quality possible. The proportion of this food may be slowly increased, until, when the child has twenty teeth fully formed, the milk diet may be safely abandoned. In the country, however, where good milk may be readily obtained, there is no doubt that it should constitute an important article of food *through childhood.* In cities this is often impracticable.

OBJECTIONS TO THIS MODE OF FEEDING.

But doubtless the objection will be made that this proposed mode of artificial lactation involves much care and expense. This is not denied. The excellence of our plan is not that it costs nothing, but that it well repays the parent for all the labor and money expended. The plan is proposed to those who wish to rear healthy and vigorous children to be the stay and solace of their declining years; and it is believed that no other plan will insure this result. And in rightly estimating the labor and expense involved in this plan, we must not forget the labor and expense which it saves. Is it no labor to watch day after day, and night after night, at the bed-side of a sick and dying infant? Is there any heavier labor, any sadder care, any greater weariness, than are involved in the months and years of painful watching over a succession of infants that languish, pine and starve, from birth to death? How many mothers feel the full force of this appeal? How many, now childless, have two, three, four, or even more, infants in little graves? And how many more mothers are there, who, with constant assiduity and care, have nursed their little ones through the many diseases of infancy, to find them growing up feeble and suffering, to languish through childhood, and to die in the very flower of their age? And how much money is yearly expended in securing medical aid for children who need not *medicine* but *food*? How often does the kind physician mournfully confess that he can do nothing for the child; that the suffering and weakness are the consequences of insufficient nourishment, and can be relieved only by such food as will strengthen the body and promote its natural and healthful development.

To those, then, who desire the welfare of their children, we propose this mode of feeding, confident that, if fairly tried, it will not disappoint their hope. Wherever it has been employed with care and the observance of the directions given, it has been successful. But let no one suppose that any of this care is excessive; that any of these directions may be safely disregarded. If full success is desired, all these precautions are requisite. The milk must be of the prescribed quality; the dilution and sweetening must be accurately and carefully performed; the hours of feeding must be regular, and the most scrupulous cleanliness observed. With these precautions, a good result may be fairly expected. If the child be not diseased, he will thrive and prosper. His teeth will show themselves in due season and without suffering. His growth will be regular and symmetrical; his functions will develop themselves according to the established order, and his infancy will be a season of high enjoyment.

Believing this to be the ordinary and natural result of this food, we most earnestly commend it to mothers whose infants are insufficiently or improperly fed. The results of this artificial lactation, properly administered, will gladden the hearts of parents and friends. If food is needed, and can be supplied, why should a child be allowed to starve?

### SHOULD MOTHERS NURSE THEIR CHILDREN?

We cannot close this little work without furnishing a reply to the question which arises in the minds of many of our readers: "If all this be true, should mothers nurse their children from their own breasts, or should they use this artificial food?" We cannot answer this question without ranging mothers in several classes, according to their milk-producing powers.

In the first class we will place those healthy and vigorous women who can fully supply their infants from their own breasts. Would that they were multiplied a hundred fold. To them we would say: "Be thankful that you can furnish to your little ones this divinely appointed food, and do all that you can to preserve this precious privilege. By constant regard to all the rules of health, seek to maintain unimpaired your present vigor. And be not weary of your work. Do not, from self-indulgence or from any improper motive, withhold from your children this food, before it is best *for them* that it should be withdrawn. Let not the allurements of pleasure nor the demands of society seduce you from your proper work, your manifest duty. And do not, by remissness in the care of your children in other respects, incur the risk of neutralizing or destroying wholly or in part the good effects of this abundant supply of wholesome food. Be thankful for the blessing, and let it be well improved." [Note C.]

In the second class we would place those mothers apparently vigorous and healthy, whose supply of milk is entirely inadequate to the proper nourishment of their children. To this class we scarcely know what to say. If it could be known that their milk, though scanty was good, it would be well for them to give it to their children, and to make up the deficiency by using the food we have recommended. We confess that we have some doubts as to the quality of this scanty secretion, and we are not sure that it is safe to give it to an infant. It is to be hoped that further researches will throw some light on this subject.

Our third, and unfortunately by far the largest class is composed of those mothers who, either from actual disease or from general debility, furnish very little milk or none at all. To them we would say:—"Why should you attempt to perform impossibilities, in which you *must* fail, when you know that the failure will bring such sad results upon your wretched children? Give to your little ones this food we have presented to you, that 'they may live and not die.' They have received but scanty nourishment from you while yet in the womb, they cannot prosper on anything that your weak bodies can furnish. Give them now an opportunity to repair the injuries already received, and to obtain health and vigor from better sources."

A serious objection at once presents itself, and we must consider it before we go further. It comes from those who fear that if the flow of milk shall cease, a second pregnancy will soon follow. Now, this *is* an objection—one worthy of serious consideration. There can be no doubt that to a feeble woman, the birth of a child every year *is* a serious matter. For her own sake and for that of her children, it would be better that the interval between successive births should be at least two years. It *is* difficult to take care of a young infant and at the same time of an infant only one year older.

Let us first consider the probable influence of the milk of this diseased or feeble mother upon her child. If this milk were harmless and only deficient in quantity, we might advise her to give it to her infant, and to use at the same time enough of our artificial human milk to make up the full amount required. But we are satisfied that *good milk* is not furnished by diseased or feeble women. Dyspepsia, in every form, is fatal to the production of *harmless* milk. If, therefore, such a mother nurses her child, *the child must suffer.* Even in cases where the mother furnishes only one-fourth of the quantity taken by the child, and where the other three-fourths are of excellent quality, we believe that the child will suffer. That is to say, we believe that such mothers give *hurtful* milk, and that it is better for the child *not to have it.*

Let us proceed a little further. Suppose that this mother, in opposition to our advice, persists in nursing her child. She will give milk for nine months perhaps, and then the flow will cease, and another pregnancy will succeed. The second year of the child's life will probably be one of almost constant sickness. "Teething," with its intestinal irritations, its diarrhœa, fever and convulsions, will bring pain, and languor, and restlessness to the child, and anxious and weary watchings to the mother. In a large number of cases, the death of the child will take place during this year. And if this sad result be averted, it will be only after much labor and anxiety, and well-grounded fear.

And how fares the second child amid these scenes of suffering? The feeble mother, worn out by anxiety and constant, sleepless care, is scarcely able to sustain her own individual life, and utterly unable to nourish fully this little one in the womb. His growth cannot fail to be hindered—the probability is great that he will not survive his birth, or if he does, it will be to drag out a few weary, suffering months, and then find rest in death. There is no exaggeration in this sketch. No reader need look far to find cases where the sickness and death of infants in one sad unbroken succession, occupy nearly the whole of the first ten or twelve years of married life.

Our advice, then, is, that when a feeble mother has an infant, she should not attempt to nurse it herself. She should do the best she can for this child that she *has*, and not cause it to suffer for fear of uncertain future events. We would say to her: "Do not sacrifice the well being of this child to your fears of a speedy pregnancy. Your child may die, your husband may die, you yourself may die; in any one of which events your fears will not be realized. Rear this child as well as you can, contented to leave the issue in the hand of God. You will not find it so difficult or so expensive to rear one healthy child a year, as to watch over one that languishes and dies in spite of close attention and medical skill. And it may be that you will have the happiness of seeing your children thrive and prosper through childhood and youth, and enter with strength upon the active career of adult life."

Another objection with many is the difficulty of carrying into effect this plan of artificial lactation. To this objection there is one simple and conclusive reply: "There is no alternative. If you adopt any other plan (except in those rare cases in which a good nurse may be obtained), you will not have good results. Your child may not die, but he will not thrive. If you are willing to count your own labor and care against the well-being of your child, you can do so. But remember that you will have labor and care, and anxiety, and fear, and perhaps death too, if you adopt an inferior mode. Take your choice; we have set the facts before you; the decision rests with you."

A third objection comes from those who live in cities, and would willingly do what they can to provide for their children. They know not how to obtain such food as we have recommended. This objection is a serious one, but it might soon be removed. Let a demand arise for this food in the cities, and it would be supplied. If meats, and vegetables, and eggs, and butter, and fruits can be sent from the country into the cities to feed the adult inhabitants, why might not this needed food be supplied for the city infants? We might then hope for some serious diminution of that fearful mortality among infants against which medical skill seems of no avail. It is famine that sweeps away these myriads of little ones; they die for want of food.

### Note A.

Schedule showing the Dilution of Milk at various Ages.

| | | | | | | | | | | | |
|---|---|---|---|---|---|---|---|---|---|---|---|
| For a child from | 2 | to | 10 | days old, | 1¼ | gills, | 3¼ | gills, | making | 4½ | gills |
| | 10 | to | 20 | " | 1¾ | " | 4¼ | " | " | 6 | " |
| | 20 | to | 30 | " | 2½ | " | 6 | " | " | 8½ | " |
| | 1 | to | 1½ | months, | 3 | " | 6¾ | " | " | 9¾ | " |
| | 1½ | to | 2 | " | 3½ | " | 7 | " | " | 10½ | " |
| | 2 | to | 2½ | " | 4 | " | 7½ | " | " | 11½ | " |
| | 2½ | to | 3 | " | 4½ | " | 7½ | " | " | 12 | " |
| | 3 | to | 3½ | " | 5 | " | 7½ | " | " | 12½ | " |
| | 3½ | to | 4 | " | 5½ | " | 7½ | " | " | 13 | " |
| | 4 | to | 4½ | " | 6 | " | 7½ | " | " | 13½ | " |
| | 4½ | to | 5 | " | 6½ | " | 7½ | " | " | 14 | " |
| | 5 | to | 6 | " | 7 | " | 7 | " | " | 14 | " |
| | 6 | to | 7 | " | 7½ | " | 6½ | " | " | 14 | " |
| | 7 | to | 8 | " | 8 | " | 6 | " | " | 14 | " |
| | 8 | to | 9 | " | 8¼ | " | 6 | " | " | 14¼ | " |
| | 9 | to | 10 | " | 8½ | " | 6 | " | " | 14½ | " |
| | 10 | to | 11 | " | 8¾ | " | 6 | " | " | 14¾ | " |
| | 11 | to | 12 | " | 9 | " | 5½ | " | " | 14½ | " |
| | 12 | to | 15 | " | 9½ | " | 5¼ | " | " | 14¾ | " |
| | 15 | to | 18 | " | 9½ | " | 5 | " | " | 14½ | " |
| | 18 onward | | | " | 10 | " | 5 | " | " | 15 | " |

It will be well to have a cup holding a gill when full.

Eight ordinary tablespoonfuls equal one gill; six equal three quarters of a gill; four equal half a gill; and two equal a quarter of a gill.

NOTE B.

The anterior fontanelle is the soft place in the front of an infant's head, just above the forehead.

NOTE C.

There is a class of mothers who give a great, even an excessive quantity of milk, who are not vigorous themselves, and whose nurslings do not thrive. The probability is that these women furnish a great quantity of milk of very poor quality. In some cases the infant is not able to take all the milk furnished by the mother, and yet it seems unsatisfied. We would advise such mothers to diminish the amount of liquid food or drink. Most, if not all, of such women drink a great deal of gruel, or beer, or some other liquid. The probable resemblance of such milk to that of swill-fed cows may, perhaps, be profitably suggested. An abundance of *good food*, well digested, can not be dispensed with by a good nurse. The infant can take only two quarts of milk daily; let it be of the best quality.

---

### APPENDIX.

### WHAT IS LACTATION?

Food is necessary to the growth and development of plants and animals. Generation, then, is not merely the quickening of a germ; it requires for its success, a supply of material to be appropriated by the germ, in building up its proper organization. In most animals, the yolk furnishes a limited amount of nourishment, usually exhausted before the young animal has reached that stage of development at which he can use the ordinary food of his race. This deficiency of the yolk-food is supplied in various ways in the various classes of animals.

Insects furnish to their young very little yolk-food. The larva is very far from the perfect development of his kind; but, having a powerful digestive apparatus, and finding food near, he eats, and thus obtains materials for further growth. Having done this, he becomes a pupa, re-entering thus the embryotic state, and there completing his development, comes forth the perfect insect.

We have said that he finds food near. This may occur in different ways. The silk-moth lays her eggs upon the mulberry-tree ; the larva feeds upon its leaves. He eats enormously, sheds his skin several times, attains at length his full growth, spins his cocoon, and then in the stillness of apparent death, carries on those further changes, for which ample materials have been secured during the larva state.

The bee, on the other hand, lays up in cells an abundant provision for the larva, by which he is carried through his later transformations.

Many insects deposit their eggs in the water. The larva there finds himself surrounded by smaller organisms, which he devours. The larva of the musquito is an example of this mode of nutrition. Having passed through the necessary changes, he leaves the water, spreads his wings, and thenceforth lives by sucking the juices of plants and animals.

In the frog, the yolk-food is very small, and the tadpole, a little fish-like animal, swimming by means of fins, breathing by means of branchiæ, is very unlike the air-breathing, four legged, leaping, insect-catching parents. He is in truth a reptilian larva; he eats largely, and grows rapidly ; without becoming torpid and inactive, he undergoes great changes of structure, fitting him for his future mode of life.

Many birds leave the egg in a very helpless state ; unable to walk or fly, they are dependent, for a longer or shorter period, on parental care. The parents supply them with worms, grubs, insects, etc. Some birds feed their young by vomiting into their open mouths half-digested food.

The gallinaceous birds here deserve especial notice. Many of them leave the egg in a state of entire independence of the mother for food. The yolk-food has in these cases sufficed for the entire development of the bird to this advanced stage.

The eggs of insects are deposited in various localities; some left on trees, some buried in the earth, some dropped in the water, some carefully deposited in cells. There is one other mode which must now be mentioned. The bot-fly deposits her eggs upon the legs of horses, so that they may be licked off, and thus carried into the stomach; there, under the influence of heat and moisture, they are quickly hatched, and the larva being provided with hooks, fixes himself firmly to the walls of the stomach, in which situation he lives by endosmosis. Having thus accumulated a sufficient amount of material for further development, he lets go, and is carried along with the refuse food, and discharged from the body of the animal, to undergo his further transformations in a different locality.

Now, in the case of this insect, there is a striking analogy with the second stage of mammalian development; the egg leaves the ovary, but instead of being laid upon the earth, or in the water, a warm place is provided for it in the midst of a highly vascular organ. Its yolk-food is soon exhausted, but not before another mode of supply has been found. It has no hooks, with which to fasten itself to the walls of its new abode, but it is upheld by the decidua, until other arrangements are made. The villous prolongations of the chorion increase its absorbing surface; and here for various terms, in various animals, it grows by endosmosis from the mother's blood. Various modifications of this process are found, and various degrees of development are thus attained. In no case, however, does it proceed so far as in the gallinaceous birds; no one of the mammalia is at birth independent of the mother for food.

To those who have observed the various domestic aniamls, this difference of development at birth is familiar; the calf, the colt, the pig, the kitten, the rabbit, the rat, are well-known instances. But we have not yet reached the lowest extreme of intra-uterine development; far below, among the implacental mammalia (the monotremata and the marsupials), we find the embryo driven forth from the womb, after a short stay, and rudely and perilously (as we should think), transferred to a new abode, and to a new mode of nutrition. We have spoken of the tadpole as a reptilian larva; here we may fairly say that we have found one among the mammalia. This embryo has just one-thirtieth of the adult length, and five hundred thousandths (.00005) of the usual adult weight. These proportions would be represented by a human embryo 2¼ inches long, and weighing 48 grains. The human embryo at three months is certainly much more developed than the young marsupial at birth. And having now done with external endosmosis, he must *drink* for his life. The nipple does not merely conduct the milk into his mouth, but forces it by muscular contraction into his stomach, deglutition being impossible in his low condition. As in his previous state, the mother's blood was brought into contact with the absorbing chorion, so now is the mother's milk, without any effort on his part, presented to the absorbents of the stomach. In view of his rudimentary state, a wonderful provision has been made whereby he still receives warmth from his mother's body; the marsupial sack contains and shelters him. Thus cherished and nourished, he is safely carried on to the independent state.

And here let us pause and look at this marsupial embryo, that we may see what lactation is. He has advanced about one-fifth of the way from conception to the independent state; four-fifths of the journey are yet before him. His sole reliance now is on his mother's milk. Not only must the milk do for him what it does for other animals, but it must perform the far more delicate and difficult work of bringing him through the intermediate embryotic condition. Here, then, in the marsupial, we see lactation in its highest manifestation. Here, too, we see plainly that it is the complement of ovarian and uterine nutrition, and that it must therefore vary in extent and influence inversely with the sum of these.

The nutrition of the germ, until it has reached the independent state, is thus seen to be carried on in four ways:

1. Simply as in the chicken, by absorption of the yolk-food.

2. The yolk food being insufficient, by means of additional nutriment furnished by the parents, as among the greater number of birds.

3. The yolk-food being very small, by additional nutriment obtained by the larva during his active term. This mode we have found among reptiles as well as among insects.

4. The yolk food being very small, nutrition is carried on by two additional processes, in both of which the nutriment is obtained from the body of the mother:

1. By intra-uterine endosmosis.
2. By lactation.

These four modes may be thus indicated:

1. The ovarian.
2. The ovarian and parental.
3. The ovarian and larval.
4. The ovarian, uterine, and mammary.

Lactation is confined to the last of these four classes. The facts relating to the other classes have been presented, that, by a wider induction, we might rise to a higher generalization, and view lactation as it appears to the general physiologist. We could not otherwise discern the exact aim and consequent extent and limits of this function, confined as it is to a few of the higher animals. Its object is now seen to be to carry on the work of development and nutrition commenced in the ovary and continued in the womb. We find it varying in extent and influence from the well-developed ruminant to the helpless, unformed embryo of the marsupial tribes.

What, then, is lactation, regarded as a function intimately connected with the development of the more highly organized animals? It is a part of the great process of generation, occupying from one to four-fifths of the entire period during which the quickened germ is dependent on the mother's body for nourishment. During its continuance, the increase in weight is from threefold to five hundred. It receives the fœtus in the condition and at the stage to which the uterus has carried him, and furnishes the materials necessary for his further growth and development.

But this is not all that lactation does. The most evident peculiarity of animal existence in the higher classes is voluntary motion and bodily activity. These demand large supplies of food. All movement in animals involves decomposition of the structures. To repair these losses, food is needed in much larger quantity than for the mere increase of the body. The ovarian and uterine life differs but little from vegetable existence. But when the young animal is driven forth from the womb, he enters upon a career of movement and activity. The respiratory movements must take place at birth, and gradually the voluntary muscles begin to act, and this action continues steadily to increase. The demand for nutriment is then much greater, during the period of lactation, than in the previous stage. The increase of the body is positively much greater, and the wants of an ever-growing activity greater still.

But we have not yet told all that lactation does. In the higher animals, the vital heat must be maintained in order to have vigor and activity. The temperature of the body must not fall below 100°, even when the external temperature is 150° lower. While yet in the womb, the fœtus partook of the mother's heat, and as there was no material increase of her own external surface, there was no necessity for any serious increase in the activity of her own heat-evolving functions. But now the young animal, with his proportionally immense radiating surface, is placed in the midst of very different circumstances. He has been introduced, perhaps, into an atmosphere the temperature of which is 75°, or 25° below his own standard. Perhaps, on leaving the womb, he finds himself floating in the waters of the frozen ocean. His heat must now be supplied from his own organization ; he can no longer depend upon his mother's body for warmth. The internal combustion began with his first breath. In order to its continuance, food must be supplied, containing oil, that, by the proper chemical union with the oxygen of the air, heat may be evolved sufficient to maintain his temperature. Without this fuel-food, the internal combustion will decline, the temperature of the body fall, and languor and death must follow.

In view of these things, we may now say what lactation is. It is the function by which food is supplied to the new-born child: food for growth; food for development; food to repair the losses caused by vital activity; food to furnish fuel for the internal combustion.

As a food-supplying function, therefore, it is the most efficient in the whole course of the generative process. No such demands are made by the fœtus on the mother's energies during the previous stages. Materials for growth are almost all that he needs until birth. But from this time onward until able to use the ordinary food of his kind, he looks to the mother for that nourishment which will enable him to maintain his temperature, to move, and to act.

The organs of this function are the mammary glands They are found under many varied forms, from the cæcal follicles of the ornithorhynchus to the elaborate and complex mamma of the higher animals.

These organs have an intermittent functional activity. It does not at first commence until the termination of the previous stages of the generative process. It continues until its work is finished, or until another generative process begins.

The product of this mammary secretion is milk. Let us examine its physicial and chemical qualities, that we may see its fitness to perform the great work of nutrition.

First, it is liquid, and can therefore be drawn from the gland in such quantity as is needed at the time. Its liquidity is indispensable, too, that it may be received by the young animal and swallowed. In some animals, it must, in addition, be injected by muscular contractions into the stomach of the young.

Second, its temperature is that of the body of the mother, and consequently it has no chilling influence upon the stomach of the child.

Nor are its intimate structure and chemical constitution less suited to the wants of the young animal. Of these wants, the first in order of time is warmth. The radiation from his relatively extensive surface would soon lead to fatal results were there not a constant evolution of heat within. The milk supplies this fuel-food in such abundance that it is not usually all consumed. Accumulations of fat, the indices of this excess above the immediate wants of the young animal, commonly take place. His genial warmth and vital activity indicate the same sufficiency. Accurate observation shows that the temperature of the blood is fully maintained.

The ncxt want of the young animal is material for growth and development. This material, needed from the moment of impregnation, has been previously derived first from the yolk-food, and afterwards from the mother's blood. It is evident that the yolk-food of the chicken must contain all the elements necessary for the formation of the body of the bird; the germ, inclosed in the shell, being precluded from all possibility of gaining any other supply. The blood of the mother contains all the elements of her own body, and consequently all existing in the body of the child, and required for its growth and development. The embryo is thus placed, during the intra-uterine period, in close and constant relation with a liquid rich in all the elements of its organization. These elements are at least sixteen in number, viz., oxygen, hydrogen, carbon, chlorine, iodine, fluorine, sulphur, phosphorus, silicon, potassium, sodium, calcium, magnesium, iron, and manganese. But not only are the elements there, but the organic combinations too, such as the fœtus requires: albumen, globulin, fibrin, hæmatin, margarin, olein, lecithin, cholesterin, serolin, chlorides of sodium and potassium, phosphates of lime, magnesia, potassa, soda, iron, and manganese, fluoride of calcium, and other most useful combinations. Thus placed in the midst of such supplies, the fœtus may well appropriate all the materials that he needs. But the time comes at length for a great change —the fœtus must be driven forth, and his contact with the mother's blood ceases from that hour. And yet his organization is incomplete; his structure is by no means finished. Let us turn to the case of the young marsupial, where the placenta did not exist, and where this uterine endosmosis has lasted only a few days. This rudimentary embryo, now in the marsupial sack, can no more dispense with the sixteen elements of his body than can the fœtuses of other animals during the latter part of their intra-uterine abode. The *blood* of the mother has done but little for him; her *milk* must do the rest, and, constituted as it is by Divine wisdom to suit his need, the young marsupial in all his feebleness is safe. No element of his body is wanting in this wonderful secretion, and forced as it is by muscular contraction into his stomach, rich in all the materials of growth, his progress is rapid to the independent condition. Thus, then, from the necessities of the marsupial we might infer the general composition of milk. But the facts ascertained by chemical analysis far transcend all our conceptions thus acquired. Not only do we find all the elements, but many of them in the same combinations as in the body and blood of the child. Four protein compounds, containing oxygen, hydrogen, carbon, azote, and sulphur, several oils, and more than ten mineral salts, enter into its composition. And what is more remarkable still, one of these salts (phosphate of lime) is found in such combination with these protein compounds, that though itself insoluble, it may be readily absorbed. This union of phosphate of lime in large proportions with casein, with albumen, and with albuminose, is one of the most important and interesting facts in relation to the composition of milk. Indispensable as it is in the formation of teeth and bone, it is thus, notwithstanding its insolubility, freely introduced into the system in an available form.

LECITHIN AND ITS RELATIONS TO THE PRODUCTION OF VITAL HEAT.

Ten oily substances are found in the milk of the cow. They constitute the butter. They are olein, butyrin, caproin, caprylin, caprin, myristicin, palmitin, stearin, butin and lecithin. We do not know the precise and special use of each of these oils. Whether they result from original differences in the various articles of food, but subserve the same purpose in the body, or whether they are fitted to supply various and peculiar wants of the young animal is not known.

One of these substances, however, deserves and must receive more careful consideration. It has long been known that the nervous system has a peculiar constituent, a phosphorised oil. Its origin in the young animal was discovered by Gobley, who found it in the yolk. It has been found in human blood in the proportion of five ten-thousandths (0.0005); and in the blood of pregnant women ninety ten-thousandths (0.0090), and in the blood of the umbilical artery seventy-five ten-thousandths (0.0075). This oil, being the special constituent of the nervous system, is furnished by the blood for the functional use of the system and for its growth. It would therefore soon be exhausted unless a supply from without were obtained. In the event of such a failure, the most serious consequences might be expected. Not only would the nervous system cease to grow and thus lose its relation to the rest of the body, but its activity would decline, and consequently the energy of all the functions. With a failure of nerve-force would coincide a failure of circulation, of heat, of digestion, of absorption and of secretion. Indeed every vital process would suffer from the deficiency of this energizing agent. And the evil would go on increasing. A weakened digestion would lead to a still further diminution of the already deficient energy, and thus by mutually depressing interactions, the decline would continue until the termination of life from inanition.

But these serious and fatal results cannot occur in a case of normal lactation. The needed lecithin is furnished by the milk in sufficient quantity to supply all the demands of the system. In cow's milk the lecithin forms three thousandths (0.003) of the whole. A calf obtains from twenty pounds of milk (his usual daily allowance) about one ounce. An infant receives about two pounds during his first year. This amount, when compared with the quantity existing in the nervous system, is very great. We see then that it must be for the most part expended in nervous action, not in the growth of the nervous system. And who that knows the almost incessant activity of young animals (when awake) can wonder at this large consumption?

CONDITIONS OF CALORIFICATION.

What are these conditions? They are: 1st. Good air, i.e. air containing a due proportion of oxygen. 2d. Good blood, i.e. blood containing a sufficiency of combustible material. 3d. A sufficiency of good air in the pulmonary cells. 4th. A sufficiency of good blood in the pulmonary capillaries. These four conditions are indispensable to full calorification. If the air be deficient in oxygen, or be introduced in too small quantity, it will be in vain that the other two conditions exist. And if there be not enough of fatty matter in the blood, nor a sufficient impulsion from the heart, the former conditions will be of no value. Two of these conditions are chemical, relating to the composition of the air and of the blood; the other two are mechanical, relating to the performance of the respiratory and circulatory acts. These last are under the influence of the nervous system, and will vary with the nervous energy.

If then, heat is to be evolved in sufficient quantity, we must have not only enough oxygen in the air, and enough oil in the blood, but we must have a supply of nervous power to induce free respiration by means of muscular action, and strong contractions of the heart, that the blood may be forced rapidly and readily through the capillaries of the lungs. With full respiration and active circulation, a sufficiency of oxygen will be mingled with the blood to furnish an abundant supply of caloric. With deficient nervous power, this vigorous action of the muscles of respiration and circulation is impossible. All pathological observation settles this point. It is a wonderful fact that butter contains not only the fuel, but the material necessary for its proper combustion. It is thus a self-regulating article. If the supply of fuel is small, the consuming power is proportionally reduced. With an increase of the consuming power, coincides a proportional increase of the material to be consumed. From these facts we may readily understand how a child may be fat and yet be deficient in strength and in vital heat. Starch-fed children are often fat, and yet are languid and weak. The starch has been digested, but as it contained no azote, it could not nourish the tissues of the body; having no phosphorus, it could not supply the wants of the nervous system. It has thus failed to produce heat or general energy. Not so with butter. The lecithin excites the nervous system to efficient action, and all the functions feel its influence. And thus the butter-fed child is often less fat than one fed on starch; but in all that constitutes physical well-being, he is far in advance.

To promote calorification, there is no equal to butter among the articles of food usually given to infants. It is probable that no substitute for it will ever be found.

INTESTINAL IRRITABILITY.

The food of infants should be all absorbed. During the fœtal state, the food of the body is obtained by endosmosis from the mother's blood. During the period of lactation, it comes from the mother's milk. In this milk *there is no refuse matter; all is absorbed and passes into the circulation.* No portion of the milk passes from the stomach through the intestinal canal to the rectum. The feces of infants are the excretions of their intestinal canal and its various glandular appendages. The lower part of the canal may thus be considered as the excreting duct of the liver, pancreas and glands of the mucous surface.

What wonder, then, that this duct should be irritated by the presence and passage of foreign bodies (lumps of curd, starch-granules, etc., etc.), and that this irritation should continue so long as these foreign bodies remain in contact with its surface?

No fact is more interesting and important (in the medical care of infants) than this, that persistent and exhausting diarrhœa often ceases at once when suitable food is given. By suitable food we mean food that is all absorbed. Three or four hours after the first dose of such food, the diarrhœa often ceases entirely.

Bearing this in mind, let us never expect health so long as foreign substances are found in the feces of infants. Even small and smooth particles of curd produce great irritation, and, in many cases, the ingestion of food produces almost immediately a discharge of mucus holding in suspension these offending substances.

DRY SOLIDS.

It thus appears that during his first year, the child receives from 110 to 143 pounds of *dry solids*. He may thus readily gain 15 or 20 pounds in weight (implying less than three pounds of dry solids), and yet have a large residue (from 107 to 140 pounds), to be expended in the production of heat and in the activity of an energetic vitality. A child thus nourished can make teeth and bone without difficulty; his functional activity need never be suspended for want of material; atmospheric changes may be successfuily resisted, and zymotic diseases will have little power.

And where, in the whole range of animal existence, will you find a more beautiful object than a vigorous, healthy child? Look at his deep and peaceful sleep; see the bright sunshine of a pleasant dream upon his gentle face. Look at him as he awakes. Listen to the sweet sounds he makes for his own pleasure, or to attract the notice of those near. And if he should at last break out into loud cries, see how quickly all traces of sorrow pass away and bright smiles replace them when his mother comes. See the eagerness with which he takes his food; the intense earnestness with which he clings to the abounding breast, and the full and deep satisfaction when this want is supplied. And when able to play, how loud and merry his laugh, how joyfully he receives caresses, or rides upon the knee, or springs in the arms of the parent or the nurse. There is no happier animal than a healthy child; nor is there anywhere to be found a more regularly operating and uninterrupted harmony of the vital functions. The beauty and energy of the outward frame are not more striking than the symmetrical development of the mental powers. The close and careful and patient observation, the cautious experiment, the unsuspecting credulity, the prying inquisitiveness, who has not beheld with admiration and delight?

This is infancy under the influence of favorable physical conditions. But alas! how few children among us progress thus steadily and rapidly during their first two years! How commonly do we associate with infancy the ideas of sleeplessness and fretfulness, and all manner of gastric, intestinal, and nervous disorders! Why is it that "*teething*" does not mean the steady, silent, unnoticed development of the teeth, but salivation, and fever, and diarrhœa, and convulsions, and death? It is not pretended that there is never any other cause than insufficiency of proper food, but there is reason to believe that four-fifths of the sufferings of infancy arise from this source. And how can it be otherwise? Look at the mothers, and say how many of them can give daily three or four pounds of milk to their nurslings. How many can furnish 110 or 140 pounds of dry solids in the first year? How many can satisfy, can fully meet the demands of even a feeble child? *A strong, vigorous, fat woman* almost always loses weight while nursing her child. The milk draws away more than the stomach of such a woman can replace, and the balance is taken, by absorption, from her previous accumulation.

And here listen to an important practical remark: A woman loses in an ordinary parturition not more than 20 pounds, containing less than 3 pounds of dry solids. This amount furnished in nine months is at the rate of 4 pounds of dry solids a year. Many women fail to furnish fully even this small amount; the infant at birth is small and meagre, looking like a starveling. Is it to be expected that such a woman will make a successful nurse? If unable to furnish that small amount, how can she supply thirty or forty times as much?

The truth is, that a woman in fully nourishing her child, must furnish as much milk in proportion to her weight as a good cow. A woman weighing 130 pounds will give daily 4 pounds of milk, containing about 5 ounces of dry solids; the cow, weighing six times as much (780 pounds), will give 20 pounds of milk, containing 30 ounces of the same solids.

It should not, then, surprise us that so many mothers fail to supply enough food for their infants. It requires physical energy and good digestion to perform this work. If we may judge by the amount of food consumed by a vigorous woman during the period of lactation, we should decide that the ordinary labor of a workingman is less exhausting than the function we are considering. Certain it is that a vigorous woman of strong digestion, while nursing a child, will eat largely and yet lose weight.

The cases in which natural lactation fail are so numerous as to excite the deepest concern. Human milk can seldom be obtained, and none of the usually employed substitutes ordinarily succeed. Is it, then, too much to hope that physicians will give serious attention and thoughtful consideration to a plan offering a substitute for human milk scientifically correct and practically successful?

